# Seedless One‐Pot Synthesis of Colloidal InAs Quantum Dots Enabling a High‐Accuracy Photoplethysmography Oximeter

**DOI:** 10.1002/advs.202524375

**Published:** 2026-04-20

**Authors:** Beom Kwan Kim, Seungin Jee, Yongnam Ahn, In‐Suh Lee, Dongeon Kim, Seongchong Park, Yujin Jung, In‐Ho Bae, Se‐Woong Baek

**Affiliations:** ^1^ Department of Chemical and Biological Engineering Korea University Seoul Republic of Korea; ^2^ Division of Physical Metrology Korea Research Institute of Standards and Science Daejeon Republic of Korea; ^3^ School of Chemical Engineering Yeungnam University Gyeongsan‐Si Republic of Korea; ^4^ KRISS School of Quantum Information University of Science and Technology Daejeon Republic of Korea

**Keywords:** colloidal quantum dot, infrared, lead‐free, oximeter, photoplethysmography

## Abstract

Near‐infrared (NIR) wavelengths offer a powerful approach for non‐invasive physiological monitoring due to the deep tissue penetration and ability to detect various endogenous molecules such as hemoglobin species, water, and lipids. Herein, we demonstrate a high‐speed, non‐contact photoplethysmography (PPG) system based on indium arsenide (InAs) colloidal quantum dots (CQDs). To this end, a seedless injection‐based one‐pot method was devised to synthesize monodisperse NIR InAs CQDs. This scalable approach enables precise bandgap tuning from 1.53 to 1.09 eV, aligning with the absorption spectra of hemoglobin and oxyhemoglobin. Furthermore, the proposed CQD‐based PPG system is integrated into a real‐time acquisition platform. Exercise‐induced desaturation tests show that the CQD‐based PPG system exhibits consistent oxygen saturation rate (SpO_2_) trends compared to commercial oximeters, exhibiting a considerable agreement of 99.76% (SpO_2_ range: 90%–92%). The system also shows reliable operation with a bandwidth up to 2.5 kHz under multi‐lock‐in detection.

## Graphical abstract

A real‐time, non‐contact photoplethysmography (PPG) system based on InAs colloidal quantum dots (CQDs) synthesized via a seedless injection synthesis is demonstrated. The measured oxygen saturation shows considerable agreement with commercial PPG devices. A dual‐lock‐in demodulation scheme enables simultaneous detection of dual‐wavelength signals within a 2.5 kHz bandwidth, highlighting its potential for advanced biosensing applications.

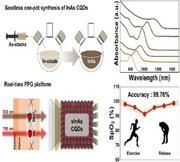

## 1 Introduction

Infrared (IR) spectra are being increasingly used in various fields such as autonomous driving [1, 2], the Internet of Things (IoT) [3, 4], and quality inspection [5]. The IR optical band is useful for biosensing and imaging owing to its optical interaction with various molecules constituting the human body [6, 7, 8].

Conventional systems used for monitoring physiological signals include the collection of samples such as saliva [9, 10], blood [11, 12], and oral cells [10, 13, 14, 15] and measurements by external stimuli [14, 16, 17, 18]. These methods have intrinsic limitations associated with visible light, which exhibits poor skin penetration and is inadequate for direct transdermal signal acquisition. While invasive and semi‐invasive procedure‐based physiological monitoring systems provide more accurate biological signals, they pose several disadvantages, including the risk of sample contamination, inconvenient procedures (blood), and the inability to facilitate continuous (saliva, oral cell) or real‐time monitoring [19, 20].

Several non‐invasive optical methods focusing on leveraging the IR spectral range, which provides high tissue penetration and minimal damage to the human body, have been investigated to overcome these limitations [21, 22]. Therefore, the development of IR semiconductors and efficient optical systems has become a key area of focus [23, 24, 25]. Among various IR materials, colloidal quantum dots (CQDs) have emerged as promising candidates owing to their facile bandgap tunability via quantum confinement, which can selectively detect various biosignals [23, 24, 26, 27, 28, 29, 30, 31].

Several CQD‐based biosensing platforms have been proposed, including Förster resonance energy transfer systems [32], saliva‐based material detection [21], photoplethysmography (PPG) [25, 33, 34] systems, and vascular imaging [7, 16] and cancer therapy using the near‐infrared (NIR) range [7, 8]. Most previous studies on CQD‐based PPG have focused on demonstrating CQD optoelectronics for waveform monitoring at fixed oxygen saturation levels [21, 25, 34, 35, 36]. However, the precise testing of real‐time detection during the physiological fluctuations and comparing the accuracy with that of the commercial oximeters remains lacking. Such investigations are crucial to ensuring the commercial viability of these systems [25, 34, 35].

In this study, we addressed the existing research gap by proposing an optical sensing system for oxygen saturation using IR indium arsenide (InAs) CQDs. In particular, we obtained InAs CQDs using a seedless injection process that ensures CQD uniformity and bandgap tunability while simplifying synthetic complexity relative to continuous injection routes. The resultant optical properties of CQDs are suitable for measuring hemoglobin (Hb) and oxyhemoglobin (HbO_2_) concentrations. Finally, we successfully fabricated a non‐contact CQD‐based PPG system with a signal processing rate of 2.5 kHz and achieved a SpO_2_ resolution up to 0.01% within a 1.98% error margin, comparable trends to that of commercial medical‐grade oximeters.

## 2 Results and Discussion

### 2.1 Seedless Injection Synthesis of IR InAs Quantum Dots

We devised a strategy that enables a scalable synthetic route for NIR CQDs to facilitate their deployment as PPG biosensors. Two key factors were considered in selecting the CQDs: (i) a RoHS‐compliant composition free of toxic elements (e.g., Pb, Hg) to ensure biocompatibility and (ii) a high absorption coefficient and precise bandgap tunability in the NIR range to ensure high performance. Hence, InAs CQDs were synthesized to address the aforementioned challenges.

**FIGURE 1:**
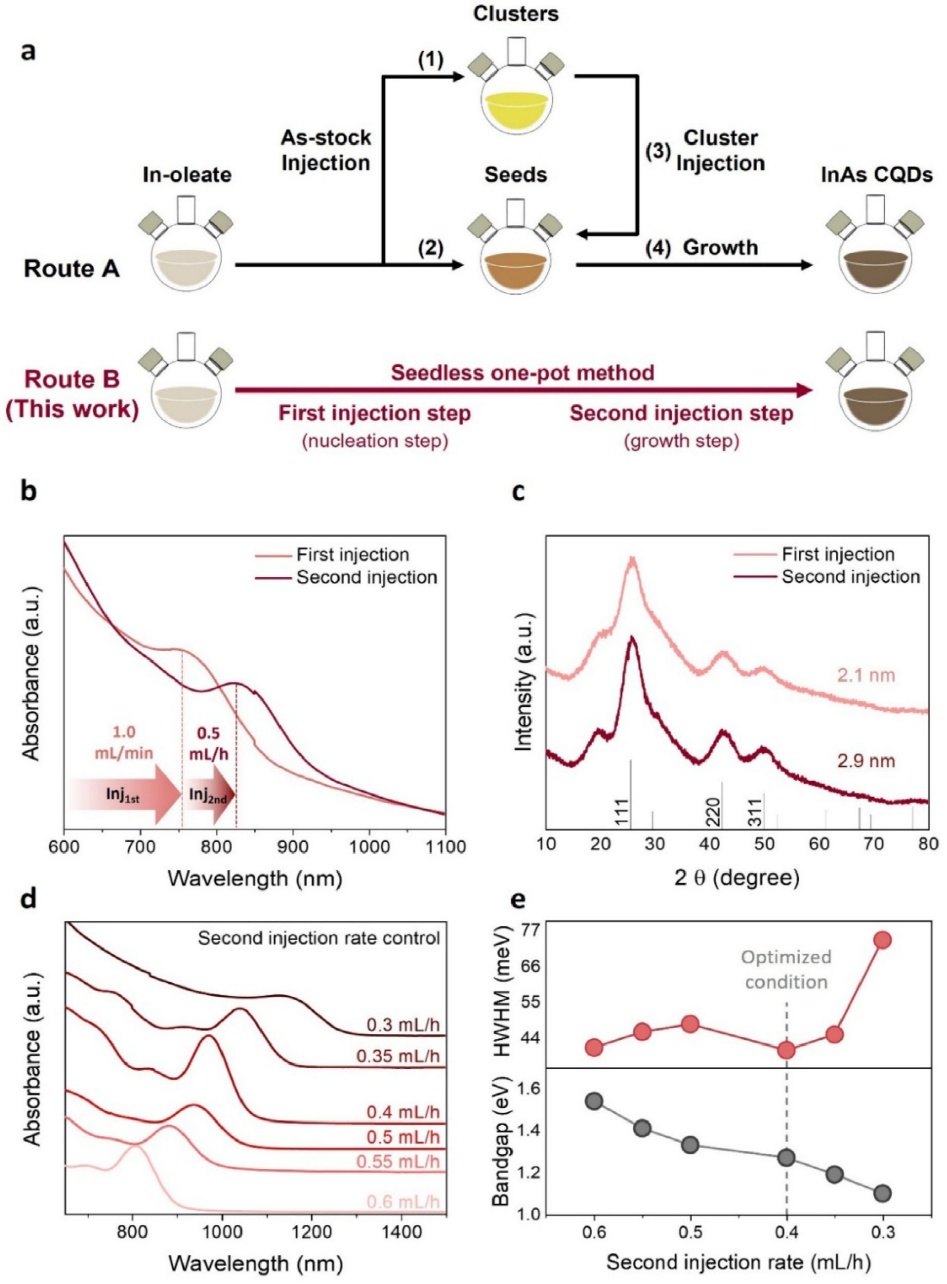
Seedless one‐pot synthesis of IR InAs CQDs. (a) Schematic illustration of Route A (continuous injection method) and Route B (seedless one‐pot method). Confirmation of the nucleation and growth steps for the seedless injection method with (b) absorbance and (c) crystallinity data. Comparison of InAs CQD synthesis via the continuous injection and seedless one‐pot methods. (d) Absorbance spectra of InAs CQDs as a function of the second injection rate control. (e) Bandgap (black) and HWHM (red) as a function of the second injection rate.

A significant challenge in synthesizing group III–V CQDs is controlling the high reactivity of group V anion precursors such as tris(trimethylsilyl)Pn [(TMS)_3_Pn, Pn = P, As, Sb] [37, 38, 39, 40], which rapidly deplete during burst nucleation, thereby hindering size‐focused growth [41, 42, 43]. Continuous injection has been proposed to mitigate precursor depletion, effectively separating the nucleation and growth stages (Route A, Figure 1a) [29, 44, 45]. Seeds and clusters are typically formed by injecting an As‐stock solution into In‐oleate solutions at different temperatures (300°C and 25°C, respectively). This process is followed by continuous cluster injection into the seed solution for controlled diffusion growth. Although this method yields monodisperse InAs CQDs, more concise and scalable approaches are necessary to facilitate commercialization in IR optoelectronics.

With this consideration, we devised the seedless injection method to obtain monodisperse InAs CQDs. Unlike conventional heat‐up or hot‐injection methods, this approach controls monomer concentration during growth through stepwise adjustment of the precursor injection rate. The reaction is performed in a single flask without pre‐formed seeds, allowing the nucleation and growth stages to be separated without additional intermediate monomers. Previous studies have demonstrated precise control over the concentration of intermediates and enabled size‐focused growth without secondary nucleation. Conversely, this study focuses on bandgap tunability with precursor conversion kinetic control according to the second injection rate [46]. The seedless injection method for InAs CQD synthesis is illustrated in Figure 1a, Route B (this study). First, we prepared a TMS‐based As‐stock solution under an inert atmosphere [47]. Subsequently, In‐oleate was prepared in the three‐neck flask with InOAc, non‐coordinated 1‐octadecene (ODE), and insulating oleic acid (OA) ligands. InAs CQDs were synthesized by injecting the prepared As‐stock into the In‐oleate solution using a syringe pump and controlling the rate to induce nucleation and growth. Compared with the continuous injection method, this seedless single‐flask route considerably simplifies the synthesis process by eliminating the separate seed‐preparation step while maintaining controlled nucleation and growth in a single flask, thereby improving process reproducibility and scalability.

We monitored the absorbance properties by controlling the injection rate to study the nucleation and growth mechanisms (Figure 1b). In the first injection step, the As‐stock was injected into a high‐temperature In‐oleate solution at a rate of 1 mL/min. An absorption peak was observed at 754 nm, similar to that in a previous study [45]. This indicates that nucleation occurred owing to the precursor conversion from the chemical reaction between the As‐stock and In‐oleate solutions. Following nucleation, the second injection rate was changed to 0.5 mL/h, and an absorption peak was observed at 824 nm after 1 h. The redshift of this absorption peak suggests that InAs CQD growth was facilitated by adjusting the injection rate of the As‐stock solution.

X‐ray diffraction (XRD) was employed to examine the growth behavior during the injection steps in more detail (Figure 1c). A broad feature around 20° was observed at the early reaction stage and is mainly attributed to the amorphous background [48]. The nuclei from the first injection step were observed at (111), (220), and (311), which characterize the diffraction patterns of the zinc blende structure [49]. This indicates that the injection rate of 1 mL/min is sufficient to generate a nucleation burst. After nucleation, crystal growth was observed in the second injection step (rate: 0.5 mL/h). During this step, we confirmed that the particle size calculated using the Scherrer equation based on the full width at half maximum (FWHM) of the (111) diffraction peak increased from 2.1 to 2.9 nm. This indicates that the As precursor, injected at a constant rate, was not depleted but contributed to nucleation and diffusion growth.

We controlled the second injection rate of (TMS)_3_As to mitigate rapid precursor conversion that hinders size‐selective growth, thereby achieving monodisperse CQD growth. Absorbance spectra showed a redshift of the first excitonic peak from 808 nm (0.6 mL/h) to 1130 nm (0.3 mL/h), indicating that controlled, diffusion‐dependent injection enables size tuning without excess monomer supply (Figure 1d). Furthermore, no significant shape transition was observed as the CQD size increased. The particles remained predominantly quasi‐spherical, as shown in Figure S1 [50].

The bandgap and half‐width at half maximum (HWHM) were plotted as a function of the second injection rate to identify the optimal conditions during the growth steps (Figure 1e). Adjusting the precursor injection rate from 0.6 to 0.3 mL/h successfully increased the InAs CQD size, decreasing the bandgap from 1.53 to 1.09 eV. A gradual increase in the HWHM was observed under secondary injection conditions of 0.6–0.5 mL/h. This suggests that at a high injection rate, the intermediates are depleted faster than the CQD growth rate and interfere with diffusion‐dependent growth [45]. By contrast, the InAs CQDs synthesized at an injection rate of 0.4 mL/h exhibited the narrowest HWHM of 52 meV, which was comparable to that of InAs CQDs based on continuous injection (∼50 meV) [45]. At lower injection rates of 0.35–0.30 mL/h, the HWHM expanded again. We concluded that at a high injection rate, size‐focused growth was limited due to the rapid depletion of the reactive precursors and differential growth rates of CQDs, whereas low injection rates below 0.35 mL/h promoted ripening at low monomer concentrations owing to side reactions that consume the reactive As‐stocks. Larger CQDs require longer growth times, during which side reactions associated with (TMS)_3_As can promote the formation of Si‐based by‐products. This broadens the size distribution and reduces the P/V ratio. Therefore, suppressing unwanted side reactions during synthesis preserves the P/V ratio, even for larger CQDs [51].

### 2.2 Optical Properties of InAs CQDs

**FIGURE 2:**
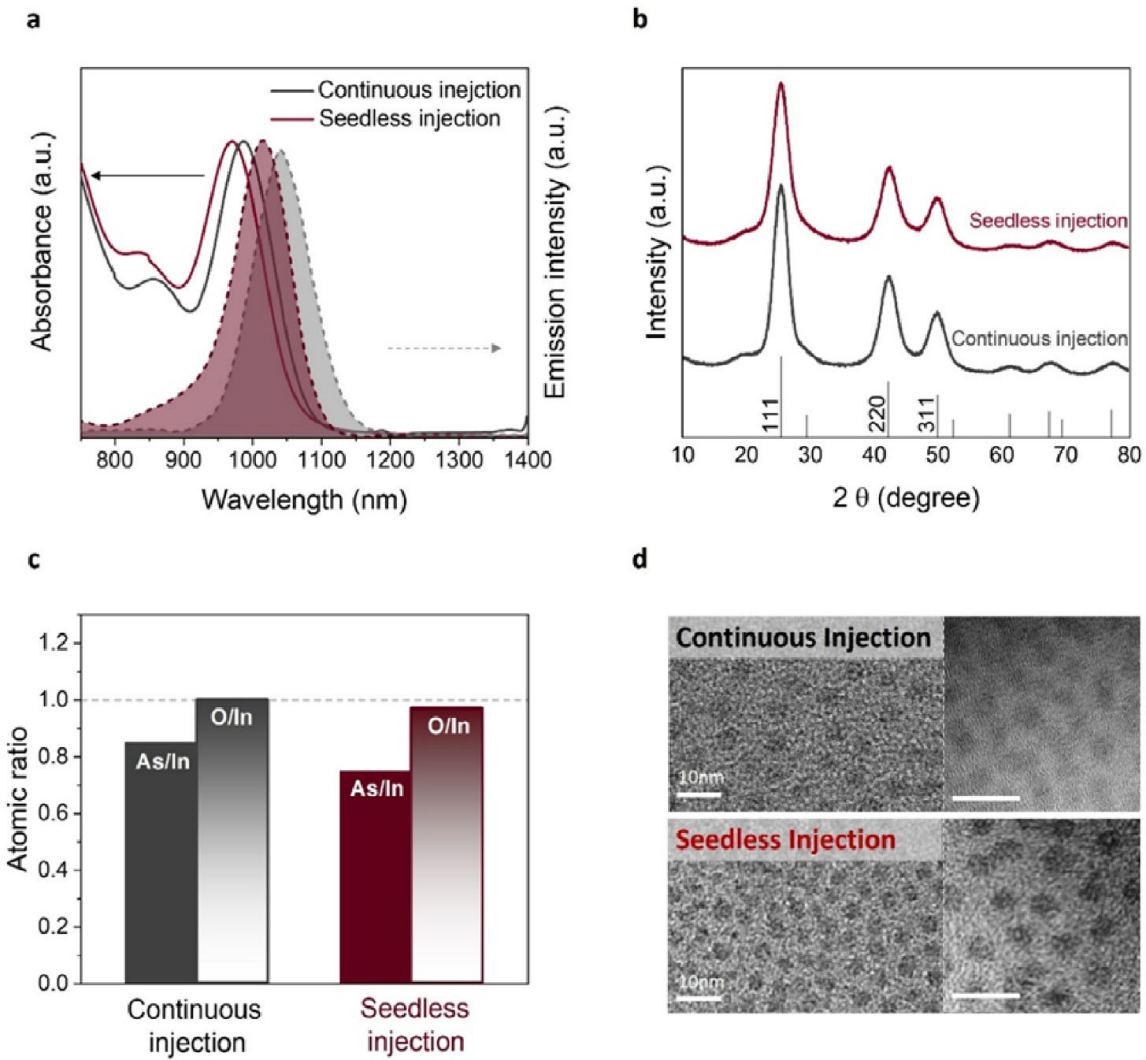
Comparison of the continuous injection and seedless injection methods. (a) Absorbance and photoluminescence emission spectra (black line: continuous injection, red line: seedless injection). (b) XRD patterns of InAs CQDs (bottom: continuous injection, top: seedless injection). (c) Atomic ratio of InAs CQDs (right: continuous injection, left: seedless injection). (d) TEM images of InAs CQDs (top: continuous injection, bottom: seedless injection). Scale bar: 10 nm.

The optical properties of the InAs CQDs synthesized by the seedless and conventional continuous injection methods were compared (Figure 2a). InAs CQDs prepared by continuous injection (Figure 1a, Route A) exhibited absorption at 970 nm and PL emission at 1026 nm, whereas seedless injection‐based CQDs (0.5 mL/h) exhibited absorption at 971 nm and emission at 1020 nm. The Stokes shifts were analogous—56 nm (continuous injection) and 60 nm (seedless injection)—indicating comparable electronic energy states. The peak‐to‐valley ratio for continuous injection (∼2.36) was slightly higher than that for seedless injection (∼1.98), and the FWHM values of emission spectra were comparable (95.7 nm for continuous and 96.8 nm for seedless injection). These results suggest that the seedless injection method achieves precursor conversion kinetics comparable to those of continuous injections. Furthermore, this approach offers practical advantages by eliminating the need for a separate seed‐growth step, thereby simplifying the synthetic route and improving its scalability.

We examined the oxidation of InAs CQDs synthesized using the two synthesis methods. XRD analysis revealed that both methods yielded well‐defined zinc blende structures with narrow FWHM, while the broad amorphous feature below 20° observed during the crystal growth stage was significantly reduced in the final purified CQDs (Figure 2b) [27]. Crystallinity trends were similar for both methods (Figure S1). X‐ray photoelectron spectroscopy (XPS) analysis revealed comparable O/In ratios for both methods (Figure 2c; Table S1), indicating that oxidation has a negligible impact on the final CQDs despite the injection of reactive As‐stock under ambient conditions.

Transmission electron microscopy (TEM) confirmed the morphology and uniformity of seedless injection‐based InAs CQDs, showing an average size of 3.8 nm (σ = 0.45) with low surface aggregation (Figure 2d; Figure S2). The size distribution is comparable to that of CQDs from continuous injection (3.9 nm, σ = 0.43), highlighting the potential for utilizing seedless injection for one‐pot monodispersed synthesis with tunable IR bandgaps.

### 2.3 InAs CQD Photodetectors for Photoplethysmography Monitoring

A broadband and tunable absorption is a useful property for non‐invasive biosensing of numerous biomarkers, such as oxidation rate [26, 34, 51, 52], blood sugar [9, 53], cancer cells [8, 33, 54, 55, 56], and platelets [55]. In particular, oxygen saturation levels are critical in various contexts, including daily activities [36], routine vital sign monitoring in patients, assessment of drug effects during anesthesia [6, 52], and as a biological signal for detecting hypoxia [6, 52]. Oxygen saturation is determined based on the absorption difference of deoxyhemoglobin and oxyhemoglobin in visible (green or red) and NIR wavelengths. Most pulse oximeters use the PPG system to detect volumetric changes in blood circulation caused by cardiac pulsations and respiratory activity [34, 36, 51, 52]. The output signals (waveform, phase, and noise) are analyzed to obtain the oxygen saturation level. For a device to be considered a medical‐grade oximeter, it is generally expected to exhibit a minimum oxygen saturation resolution of 1% with an error margin of ±2%.

**FIGURE 3:**
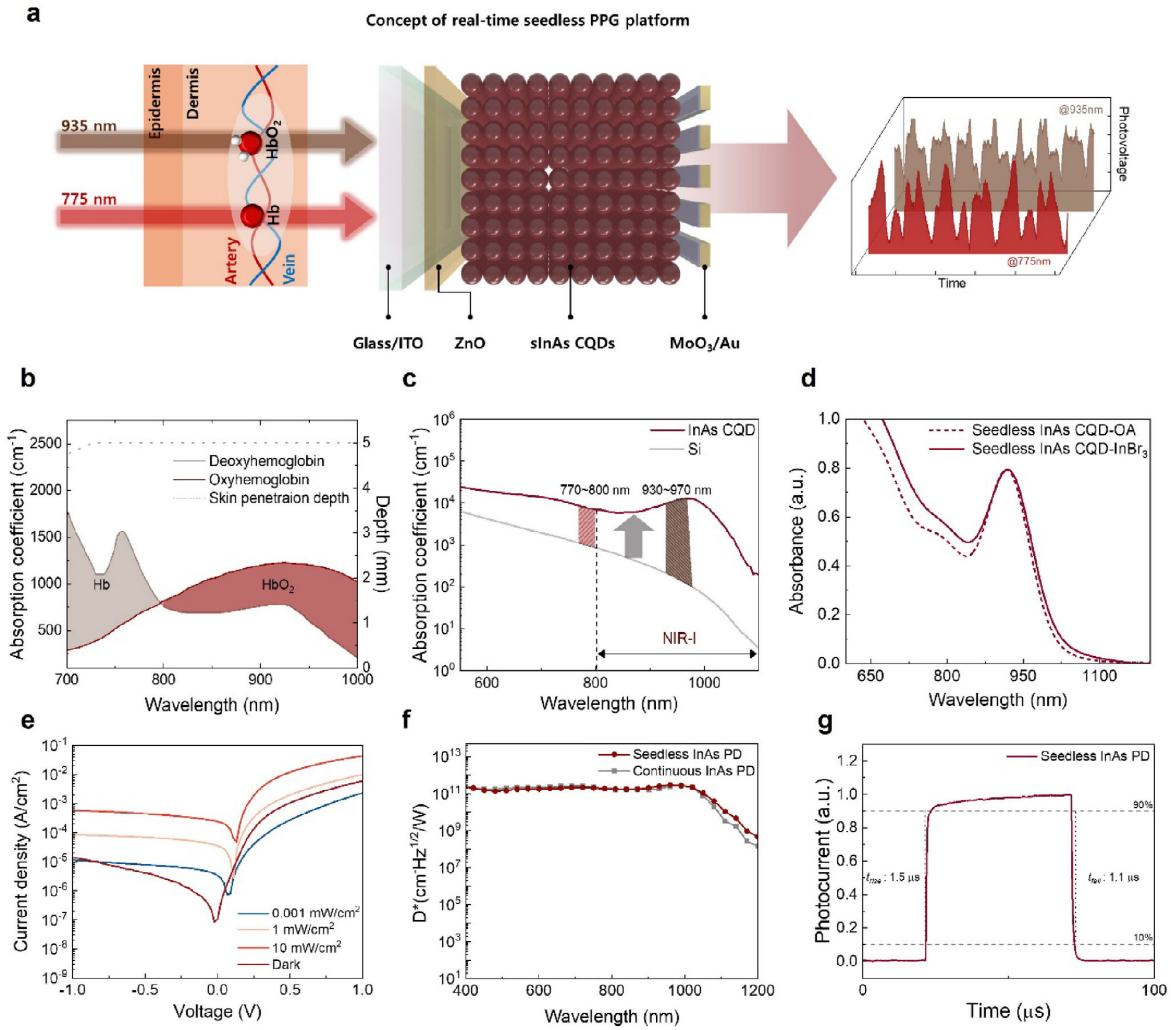
Characterizations of the InAs CQD photodetector. (a) Illustration of a PPG system with a seedless InAs CQD‐based photodetector. (b) Absorption coefficients of hemoglobin (light brown) and oxyhemoglobin (red) from the visible to NIR range, with corresponding skin penetration depth indicated on the right *y*‐axis. (c) Comparison of the absorption coefficients of silicon and InAs CQD across the wavelength range (dash‐patterned area: absorption spectrum in red, NIR region) according to the wavelength (light gray dotted line). (d) UV–vis spectroscopy of seedless InAs CQD ink before (dashed line) and after (solid line) ligand exchange. (e) *J*–*V* curves under dark (dark red, device area: 0.1 cm^2^) and one sun illumination (device area: 0.045 cm^2^) with 3 types of ND filters (ND 1.0; Red, ND 2.0; Orange, ND 5.0; Blue, respectively) (f) Detectivity comparison of photodetectors fabricated using seedless (red) and continuous injection InAs CQDs. (gray) (g) Rise and fall time of seedless InAs CQD photodetector under 935 nm laser illumination (10 kHz, device area; 0.1 cm^2^). The seedless InAs photodetector exhibits a fast response suitable for ∼100 Hz PPG signal processing.

Our proposed optical system for PPG measurement utilizes 775 nm (red) and 935 nm (NIR) wavelengths, which offer low skin scattering and low water absorption [57] (Figure 3a). Photons from the two laser diodes penetrate the skin and are attenuated by deoxyhemoglobin and oxyhemoglobin. Both resulting optical signals are simultaneously detected by the photodetector and processed in real time. Deoxyhemoglobin shows notable absorption at 775 nm (ε_Hb_ ≈ 1200 cm^−^^1^·m ^−^^1^ > ε_HbO2_ ≈ 600 cm^−^^1^·m ^−^^1^), whereas oxyhemoglobin exhibits a disticnt absorption difference at 935 nm (ε_Hb_ ≈ 848 cm^−^^1^·m ^−^^1^, ε_HbO2_ < 1216 cm^−^^1^·m ^−^^1^) confirming that the dual wavelengths used in our setup are suitable for detecting oxygen saturation levels (Figure 3b). Most commercial PPG oximeters use Si‐based photodetectors, which provide high detectivity over a broad spectral range from the visible to NIR wavelengths. However, the absorption coefficient of silicon decreases significantly beyond 900 nm [58, 59, 60,]. By contrast, InAs CQDs exhibit a high absorption coefficient of 1.30  ×  10^4^ cm^−1^_,_ approximately 100 times higher than that of silicon beyond 940 nm, an important region for oxyhemoglobin detection [6, 22, 34, 36]. Therefore, we hypothesized that the superior NIR absorption characteristics of an InAs CQD‐based photodetector may offer enhanced sensitivity [59] (Figure 3c).

Subsequently, we fabricated the InAs CQD‐based photodetector to demonstrate oxygen saturation PPG systems. Native OA ligands were exchanged with InBr_3_ as short ligands to fabricate conductive InAs CQD ink [24]. UV–vis spectroscopy of the seedless InAs CQDs shows a redshift (4 nm) consistent with that observed in the continuous‐injection process. This indicates that the surface reactivity with InBr_3_ exhibits similar ligand‐exchange characteristics, suggesting that the same passivation processes can be applied to the seedless CQDs. The shifted exitonic peak was placed at 920 nm, which is an effective NIR detecting range for the difference absorption of oxyhemoglobin and deoxyhemoglobin. (Figure 3d; Figure S4) Furthermore, Fourier transform infrared spectroscopy (FT‐IR) was performed to verify whether OA was substituted with InBr_3_. OA‐passivated InAs CQDs showed intensity peaks corresponding to CH_3_ stretching vibrations at 2800–2900 cm^−1^ and carboxyl groups at 1750 cm^−1^, which disappear after InBr_3_ ligand exchange, indicating successful surface modification [23, 24, 28] (Figure S5). Furthermore, XPS revealed that the ligand‐exchanged CQDs exhibited a higher count of Br at 68–70 eV than pristine InAs CQDs, confirming successful surface ligand exchange [24, 47] (Figure S6).

We fabricated a photodiode‐type structure, ITO/ZnO/InAs CQDs/MoO_3_/Au, using both continuous injection and seedless synthesis methods (Figure 3a) [24, 25]. The photodetector was characterized under both dark and light‐biased conditions. The control and seedless InAs CQD photodetectors exhibit similar dark current densities of 8.23 × 10^−^^8^ and 8.62 × 10^−^^8^ A/cm^2^, respectively. The seedless InAs CQD photodetector exhibits a rectification ratio of 413.79 at ±1 V, indicating well‐defined diode characteristics. Under AM 1.5G illumination with intensity control using a neutral density filter, it shows an open‐circuit voltage (*V*_oc_) of 0.132 V at 10 mW/cm^2^ (device area: 0.045 cm^2^), comparable to that of the control device (Figure 3e; Figure S7). Subsequently, we characterized the external quantum efficiency (EQE) to evaluate the spectral response of the photodetector. Under a bias of ‐1 V, the EQEs of the seedless and continuous InAs CQD devices were 24.4 and 20.4%, respectively (Figure S8). The seedless InAs CQD photodetector demonstrated a calculated responsivity of 0.15 A/W and a specific detectivity of 3.01 × 10^11^ Jones, comparable to that of the continuous method (Figure 3f; Figure S9). Furthermore, the seedless InAs CQD photodetector also exhibits comparable dark current, EQE, and detectivity to recently reported NIR InAs CQD photodiodes (Table S2).

For a real‐time biomonitoring platform, photodetectors must achieve a rapid response time to enable high‐resolution optical signal acquisition and a minimum sampling rate of 100 Hz required for medical‐grade PPG oximetry [6, 52, 53, 61]. The InAs CQD photodetector exhibits a rapid signal processing capability, with a fall time of 1.1 µs. (Figure 3g; Figure S10).

### 2.4 Real‐Time Detection of Oxygen Saturation

**FIGURE 4:**
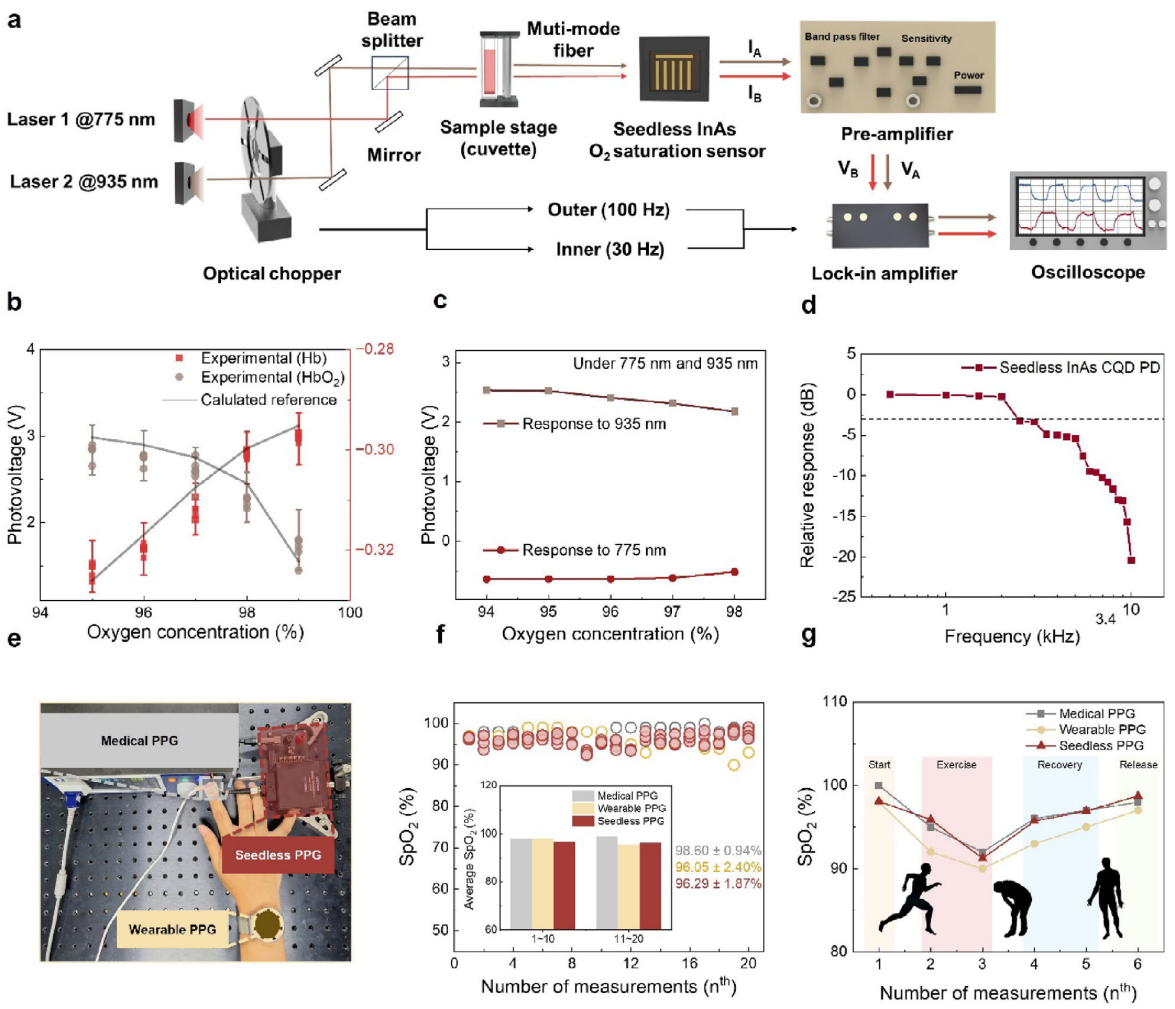
Real‐time oxygen saturation detection using a CQD‐based PPG system. (a) Overall schematic for the detection of the total oxygen saturation level. (b) Photovoltage response on the 775 nm (red) and 935 nm (brown) channels as a function of the oxygen concentration in the PBS solution (95%–99% at 1% intervals), plotted with the predicted model (gray). (c) Photovoltage signal fluctuation depending on the oxygen‐level changes for different sample conditions (95%–99% at 1% intervals) under dual (775 and 935 nm) laser irradiations. (d) Frequency‐dependent photoresponse of the proposed PPG system (dB scale) under dual wavelength (775 and 935 nm) lock‐in detection. The frequency of 775 nm was fixed at 30 Hz and swept in 0.5∼10 kHz (0.5 kHz step) for 935 nm. The output signal amplitude stably maintained to 2.5 kHz (−3 dB bandwidth). (e) Photographic image of real‐time measurements (medical, wearable, and InAs CQD‐based PPG) (f) Comparison of measured SpO_2_ using medical‐grade for reference (light gray), wearable (yellow), and InAs CQD‐based (red) PPG sensors. The inset shows the average measured oxygen saturation level for each PPG measurement. Gray, yellow, and red bars correspond to medical‐grade, wearable, and InAs CQD‐based PPG systems, respectively. (g) Response variation under four different physical conditions (start: yellow, exercise: red, recovery: blue, relax: green filled area) using three types of PPG (medical‐grade: light gray, wearable: yellow, InAs CQD‐based: red).

We examined the InAs CQD photodetectors for real‐time oxygen saturation monitoring using a dual‐laser PPG setup, as a proof‐of‐concept demonstration. We employed lock‐in detection [61, 62, 63] with optical choppers modulating the lasers at 100 and 30 Hz to overcome the issue of a single photocurrent output in a typical jig. The modulated beams were combined via mirrors and a beam splitter, coupled into a multimode fiber, and delivered to the detector through a cuvette filled with a blood‐mimicking Hb/HbO_2_ phosphate‐buffered saline (PBS) solution. A preamplifier and lock‐in amplifier extracted signals corresponding to each modulation frequency, enabling accurate multi‐signal separation. Photovoltage changes were detected in the millivolt range, ensuring high‐resolution PPG measurements (Figure 4a). Clinically, SpO_2_ values of 95%–99% are considered normal, 90%–95% are in a warning range, and values below 90% indicate hypoxemia requiring clinical attention. Therefore, it is particularly important to accurately distinguish SpO_2_ values within the 90%–100% range [35, 64, 65]. To evaluate device performance in this physiologically relevant range, we first tested the proposed oximeter using blood‐mimicking samples corresponding to normal physiological conditions.

Theoretical models were tested using the absorption coefficients of deoxyhemoglobin and oxyhemoglobin and based on the relationship between the EQE and photovoltage [23, 24, 25, 48, 66].

(1) $$V_{out}=I_{out}\;R\frac{1-e\alpha d}{\alpha d}P$$

where *V_out_* is the output photovoltage, *I_out_* is the output photocurrent, *R* is the external load resistance, *e* is Euler's number, *P* is the incident optical power, α is the absorption coefficient, and *d* is the thickness of the cuvette. Based on this model, we examined the relationship between photovoltage and the concentration of individual hemoglobin species. Two types of samples were independently prepared. We dissolve sodium dithionate with hemoglobin powder in PBS solution for deoxyhemoglobin. We prepared oxyhemoglobin by vortex this solution in air for a while and mixed it quantitatively for oxygen saturation levels ranging from 95% to 99%, reflecting physiologically relevance conditions defined by clinical guidelines [64]. To verify the quantification of our mimicking samples, we applied the Winterbourn equation [67] (Table S3). As shown in Figure 4b, the photovoltage decreased with increasing oxyhemoglobin concentration, whereas the photovoltage increased as the deoxyhemoglobin levels increased. These results align with the predicted model obtained from Equation (1), confirming that the device can effectively detect the oxygenation levels through photovoltage response (Figure 4c). Note that the low concentration of sodium dithionite and its decomposition byproducts primarily absorb in the ultraviolet region and exhibit negligible absorption in the red and near‐infrared spectral ranges relevant to SpO_2_ measurements [68, 69].

We characterized the frequency‐dependent photoresponse (0.5–10 kHz) to verify signal stability under multi‐frequency operation. For the proposed system, we measured amplitude variation of photovoltage by sweeping the lock‐in frequency from 500Hz to 10kHz under 935 nm laser illumination, while maintaining 775 nm laser at 30 Hz modulation. We achieved a −3 dB bandwidth of 2.5 kHz in our PPG system, suggest that the signal processing rate of our demonstration is sufficient for reliable oxygen saturation measurements in the proposed PPG system [70] (Figure 4d).

Based on the preceding outcomes, the SpO_2_ signals obtained from the CQD‐based PPG system was compared with those from a medical‐grade PPG sensor (NELLCOR, Medtronic, Ireland), and a wearable PPG sensor (Galaxy watch 6, Samsung, Republic of Korea). For medical and CQD‐based PPG devices, oxygen saturation was tested at the fingertip [6, 52] (Figure 4e). The CQD‐based PPG system collects approximately 3–5 data points on average within 20 ms to quantify the oxygen saturation level. The performance of the InAs CQD‐based PPG oximeter was comparable to that of the wearable PPG oximeter, with an error range from 0.83% to 1.60%. Across 20 measurements, the mean SpO_2_ was 96.29% ± 1.87% for the InAs CQD‐based PPG, 96.05% ± 2.40% for the wearable device, and 98.60% ± 0.94% for the medical‐grade device. The observed variation is attributed to the diode‐type laser source, which does not maintain a single operating frequency and gradually attenuates in power over time (Figure 4f; Figure S11). These results show that the InAs CQD‐based PPG system exhibits comparable performance to typical commercial PPG oximeters.

Considering practical use, the three different PPG oximeters were further tested during physical activity. Under dynamic physiological states induced by physical activity, oxygen saturation levels fluctuate with respiration as oxygen uptake increases the oxyhemoglobin concentration in blood vessels. Subsequently, oxygen consumption during cellular metabolism leads to the production of deoxyhemoglobin, resulting in reduced oxygen saturation levels [34, 71]. The physical activities were performed in four distinct phases: start, exercise, recovery, and rest. During the exercise phase, two sets of running were performed to elevate the respiration rate. Measurements were recorded immediately after exercise and at 1.5‐min intervals throughout the recovery phase. At the initial stage, oxygen saturation was measured as 100.0% (medical, gray), 98.0% (wearable, yellow), and 98.1% (InAs CQD‐based, red). After vigorous exercise, the oxygen saturation dropped to 92.0, 90.0, and 91.2%, respectively. During recovery, the oxygen saturation level gradually increased by 3%–4%, reaching final values of 98.0% (medical), 97.0% (wearable), and 98.7% (InAs CQD‐based), respectively. All three devices exhibited similar trends in the response to exercise‐induced desaturation and recovery. The wearable device showed a larger difference during the exercise phase, presumably due to motion artifacts and sweat‐induced signal degradation, which are common limitations in wrist‐based measurements. Stable values at the start and end phases confirmed the reliability of each device. The InAs CQD‐based PPG showed a maximum error margin of 1.98%, which is comparable with that of the medical‐grade oximeter across all phases, demonstrating high accuracy in detecting rapid oxygen saturation changes (Figure 4g). Moreover, the mean absolute error (MAE) was calculated to be 1.43%, demonstrating the highest accuracy among the various commercial smartwatches, further highlighting the overall consistency and precision of the system (Table S4).

## 3 Conclusions

In this study, we introduced a novel non‐contact PPG system utilizing InAs CQDs synthesized via a scalable seedless method. The developed system achieved reliable photovoltage signals with amplitudes across a wide dynamic range. Under dual‐frequency modulation with frequency sweeping up to 10 kHz through a 935 nm laser, it exhibited stable signal extraction throughout the operational bandwidth of 2.5 kHz. For the oxygen saturation measurements, proposal system exhibits high resolution comparable to that of commercial oximeters. Furthermore, the system achieved a high accuracy of 99.76% under exercise conditions within the tested SpO_2_ range, exhibiting similar SpO_2_ trends compared to commercial PPG oximeters, indicating its potential for robust real‐time physiological monitoring under dynamic conditions. Leveraging the tunable bandgaps of InAs CQDs precisely aligned with the absorption spectra of Hb and HbO_2_ significantly enhanced the measurement accuracy and reliability. The integration of lock‐in detection further improved signal precision and separation, highlighting the potential of the method in biosensing applications. The proposed method offers a cost‐effective and widely deployable platform suitable for diverse biomedical and wearable sensor applications. Future advancements, including integrating high‐speed optical switches and stable single‐frequency lasers, could further boost data acquisition speed, stability, and precision. These improvements will facilitate the detection of subtle physiological signals, enhancing real‐time health monitoring and diagnostic capabilities.

## 4 Methods

### 4.1 Materials

Indium acetate (In(OAc)_3_, 99.99%), indium bromide (InBr_3,_ 99%), ammonium acetate (NH_4_Ac, 98%), oleic acid (OA, technical grade, 90%), 1‐octadecene (ODE, technical grade, 90%), toluene (ACS reagent, ≥99.5%), tetrachloroethylene (TCE, ≥99.5%), dioctylamine (DOA, 97%), hexane (HPLC, ≥97.0%), hemoglobin human (lyophilized powder), sodium dithionate (Na_2_S_2_O_4_) and PBS were purchased from Sigma–Aldrich (Burlington, Massachusetts, USA). n‐octane (98+%) was purchased from Alfa Aesar (Heysham, Lancashire, UK). (TMS)_3_As (≥90%) was purchased from JSI Silicon (Seongnam‐si, Gyeonggi‐do, South Korea), and n‐butanol (BuOH, HPLC, 99.8+%) was purchased from Junsei Chemical (Tokyo, Japan). Furthermore, N, N‐Dimethylformamide (DMF, HPLC) was purchased from Daejung Chemical (Shiheung‐si, Gyeonggi‐do, South Korea).

### 4.2 Synthesis of InAs CQDs by Seedless Injection

For preparing the In‐oleate solution, 3.5 mmol of In(OAc)_3_ and 4.3 mmol of OA were mixed in 17.5 mL of ODE in a three‐neck flask. The solution was degassed under vacuum at 120°C for 1 h and heated to 300°C under Ar flow. An As‐stock solution was prepared in a glovebox by mixing 0.5 mmol of (TMS)_3_As and 1.5 mL of DOA in 1 mL of degassed ODE, followed by stirring at 60°C for 10 min. After stirring, the As‐stock solution was loaded into a syringe. In the first injection step, when the temperature of the In‐oleate solution reached 300°C, the prepared As‐stock solution was initially injected at a rate of 1 mL/min using a syringe pump. In the second injection step, after 2 min, the injection rate was adjusted to the desired rate (0.3–0.6 mL/h), and the remaining As‐stock solution was slowly injected at that rate. Following the injection, the reaction mixture was cooled to room temperature, and the InAs crude solution was divided into 8 mL aliquots in conical tubes. We added 40 mL of BuOH to each tube and centrifuged at 6000 rpm for 5 min. The precipitate was redispersed in 8 mL of hexane. Then, 12 mL of BuOH was added and centrifuged at 4000 rpm for 5 min. The precipitate was discarded, and the supernatant was collected. Subsequently, 16 mL of BuOH was added to the supernatant and centrifuged at 8000 rpm for 5 min to remove insoluble by‐products. Finally, the precipitate was redispersed in 8 mL of hexane and then 35 mL of BuOH, and centrifuged at 8000 rpm for 5 min. The final precipitate was dried under vacuum for 4 h and redispersed in octane or TCE.

### 4.3 Synthesis of InAs CQDs by Continuous Injection

InAs CQDs were synthesized using a modified method based on a previous study [45]. InAs seeds were prepared by mixing 1 mmol of In(OAc)_3_ and 3 mmol of OA in 5 mL of ODE. The solution was degassed at 120°C for 2 h under vacuum and heated to 300°C under Ar. An As‐stock solution was separately prepared in a glovebox by mixing 0.5 mmol of (TMS)_3_As and 1.5 mmol of DOA in 1 mL of degassed ODE and stirring it at 60°C for 10 min. After stirring, the As‐stock solution was loaded into a syringe and injected into the hot In‐oleate solution at 300°C. The reaction mixture was maintained at this temperature for 20 min to grow InAs seeds. For preparing the InAs clusters, 6 mmol of In(OAc)_3_ and 18 mmol of OA were dissolved in 30 mL of ODE and degassed at 120°C for 2 h. In parallel, an As‐stock solution was prepared by mixing 3 mmol of (TMS)_3_As and 9 mmol of DOA in 6 mL of degassed ODE and stirring it at 60°C for 15 min. The As‐stock solution was injected into the In‐oleate solution at room temperature and stirred for 15 min to form amorphous InAs clusters. For the synthesis of InAs CQDs, the resulting InAs cluster solution was injected into the InAs seed solution at 300°C using a syringe pump at a rate of 2 mL/h for 4 h. After completion, the reaction mixture was cooled to room temperature and purified using the same method in the seedless injection synthesis of InAs CQDs.

### 4.4 Photodetector Fabrication

ZnO sol‐gel was deposited on washed ITO/glass substrates for electron transport layer. CQD inks in DMF (200 mg/mL) were spin‐coated on the substrate at 2000 rpm for 30 s. MoO_3_ (10 nm) was deposited as a hole transport layer and Au (120 nm) via thermal evaporation. InAs CQDs with an excitonic peak at 935 nm were used for ink fabrication. The ligand exchange process was performed in an N_2_ flow glovebox. OA‐CQDs were prepared via dissolution in octane (20 mg/mL). For ligand exchange, InBr_3_ was dissolved in DMF and vortexed with pristine CQDs for 30 s. After phase transfer, the mixture was rinsed with octane for three cycles with 15 s of vortexing for each. Subsequently, 6 mL of toluene was added to the mixture and centrifuged for 90 s. The precipitated CQDs were dried in a vacuum for 15 min and dispersed in DMF.

### 4.5 Device Characterizations

#### 4.5.1 Dark Current Measurements

The current–voltage characteristics under dark conditions were measured using a Keithley 2401 source‐meter (Keithley Instruments, Cleveland, OH, USA) unit under ambient conditions. The *J–V* curves were swept from −1.0 to 1.0 V at 0.02 V increments with a 50 ms delay time at each step.

#### 4.5.2 Light‐Biased *J*–*V* Measurement

The *J*–*V* characteristics under illumination were measured on devices with an active area of 0.045 cm^2^. A solar simulator providing one‐sun illumination (100 mW/cm^2^) was used as the light source. Neutral density (ND) filters were employed to vary the light intensity from 5.0 to 0 in 1.0‐step increments, corresponding to roughly tenfold reductions in light intensity at each step.

#### 4.5.3 EQE

The EQE spectra were measured using the QuantX‐300 measurement system (Newport). Monochromatic white light from a xenon lamp (400 W) was chopped at a frequency of 220 Hz and used to illuminate the device. The spectral responses were calculated from the measured photocurrent.

#### 4.5.4 Rise and Fall Time

The photoresponse of the device was measured by illuminating a 0.1 cm^2^ area with a 940 nm light source (MDL‐NS‐940, CNI Laser) modulated at 10 kHz with a 50% duty cycle. The resulting photocurrent was recorded using an oscilloscope (DSO7054A, Tektronix).

#### 4.5.5 Responsivity Calculation

The responsivity (R) was calculated from the measured external quantum efficiency (EQE) using following Equation (2):

(2) $$R=\frac{q\cdot \lambda \cdot EQE}{hc}$$

**q** :elementary charge (1.602 × 10^−19^ C),

**λ**:incident wavelength (m),

**h**:Planck's constant (6.626 × 10^−34^ J·s),

**c**:Speed of light (3.00 × 10^8^ m/s),

**EQE**:external quantum efficiency.

#### 4.5.6 Specific Detectivity Characterization

The specific detectivity (D*) of the quantum‐dot photodetector was evaluated based on the responsivity derived from the external quantum efficiency (EQE) and the shot noise‐limited current. Detectivity is defined as following Equation (3):

(3) $$D^{*}=\frac{R\sqrt{A\;\Delta f}}{i_{shot}}$$

**R** :Responsivity (A/W),

**A**:effective active area of the device (cm^2^),

**Δf** :the electrical bandwidth (Hz),

**i** :the noise current (A).

The shot noise current is calculated by following Equation (4):

(4) $$i_{\mathrm{shot}}=\sqrt{2qI_{\mathrm{dark}}\Delta f}$$

where I_dark_is the dark current (A).

### 4.6 CQD Characterizations

#### 4.6.1 UV–vis–NIR Spectrometer

The absorption spectra of InAs CQDs were evaluated using a UV–vis–NIR spectrometer (Jasco, V‐770). The samples were prepared by diluting the solution with TCE and DMF in a quartz cuvette.

#### 4.6.2 Photoluminescence (PL)

PL spectra were measured using a Horiba Nanolog. The samples were prepared by dilution in octane.

#### 4.6.3 TEM

TEM images were acquired using a Tecnai F30 Super‐Twin TEM microscope accelerated at 300 keV. The samples were prepared using a carbon‐coated grid with InAs CQDs dispersed in hexane.

#### 4.6.4 XPS Analysis

XPS analysis was performed using Thermo Fisher Nexsa G2. A monochromatic Al Kα X‐ray source was used.

#### 4.6.5 XRD Analysis

XRD patterns were obtained using a Rigaku SmartLab device. The X‐ray generator used was a 9 kW Cu source. The samples were prepared by drop‐casting a film onto the glass substrate.

#### 4.6.6 FT‐IR Analysis

FT‐IR measurements were performed using an Agilent Cary 630 FTIR. The samples were prepared using KBr‐mixed pellets.

### 4.7 PPG Signal Processing

The experimental setup for real‐time oxygen saturation measurement involved a 935 nm laser diode (Thorlabs, DBR935PN) and a 775 nm laser diode (Thorlabs, L780P010), each controlled by separate laser diode controllers (Thorlabs, LDC 240C; Stanford Research Systems, LDC 501). The incident light from both lasers collimated using collimation lenses and transmitted through a multimode optical fiber. The beams were then directed through a sample cuvette and onto the seedless InAs CQD photodetector. The resulting photocurrent was amplified by a preamplifier (Stanford Research Systems, SR560), converted into a voltage signal, and monitored via an oscilloscope (Keysight, DSOX1204A).

#### 4.7.1 Lock‐In Detection

Owing to the limitations of the jig, which provides only a single photocurrent output and prevents the simultaneous detection of multiple optical signals, we implemented lock‐in detection to achieve signal separation. Both laser sources were modulated at reference frequencies of 100 and 30 Hz using optical choppers (Stanford Research Systems, SR540). The modulated beams were aligned using multiple mirrors, combined by a beam splitter, and transmitted through the multimode optical fiber. After passing through the sample cuvette, the combined incident light was inputted into the photodetector. The lock‐in amplifier (LIA‐MV‐150) was then used to selectively extract the components corresponding to each modulation frequency, enabling the precise separation of the signals despite the single photocurrent output limitation of the jig.

### 4.8 Hb/HbO_2_ Solution Photoresponse Detection

Hemoglobin (Hb) PBS solutions were prepared by dissolving hemoglobin powder in PBS to achieve mass percentages of 1%, 2%, 3%, 4%, and 5%. For oxyhemoglobin (HbO_2_) solutions, hemoglobin powder was dissolved in PBS to obtain a concentration of 95%–99% HbO_2_ with a small amount of 0.1 mm sodium dithionite solution. After dissolution, the solution was vortexed for 30 s with the cap removed to ensure sufficient oxygen exposure. This resulted in a color change indicative of oxyhemoglobin formation. To confirm that the samples were quantitatively mixed, the concentrations of oxyhemoglobin (HbO_2_) and deoxyhemoglobin (Hb) were calculated using the Winterbourn method as following Equation (5):

(5) $$\begin{array}{cc} \left[\mathrm{OxyHb}\right] & =-75.78\cdot OD_{560}+103.16\cdot OD_{576}-38.39\cdot OD_{630}\\ \left[\mathrm{DeoxyHb}\right] & =132.6\cdot OD_{560}+74.10\cdot OD_{576}-68.33\cdot OD_{630} \end{array}$$

where OD_λ_is the optical density measured at the wavelength λ.

#### 4.8.1 Signal Detection

Five measurements were performed each for Hb only, HbO_2_ only, and mixed concentration conditions. The resulting optical signals were observed and analyzed using an oscilloscope (Keysight, DSOX1204A) at a time scale of 20 ms.

### 4.9 Real‐Time Oxygen Saturation Detection

All measurements were consistently taken from the right hand and wrist to minimize variability. An integrating sphere was incorporated into the PPG platform to enhance signal quality and minimize ambient noise. The light source was positioned horizontally to the fingertip to maintain a constant angle of incidence, ensuring uniform illumination. In total, 20 real‐time measurements were performed using both medical and wearable PPG oximeters. For each reference measurement, the seedless InAs PPG system collected approximately 3–5 data points within 20 ms, enabling high‐resolution signal acquisition. Oxygen saturation was quantified by measuring the baseline under the empty condition of the integrating sphere and normalizing the photovoltage generated by light passing through the fingertip.

#### 4.9.1 Error Calculation

Over 20 repeated sets of measurements, the reference value for each set was defined as the mean of the measured data, and the error range was determined using the following Equations (6) and (7).

(6) $$Mean\;value=\bar{x}=\frac{1}{n}\sum_{i=1}^{n}\left|x_{i}\right|$$

(7) $$Error\;range=\pm \sqrt{\frac{1}{n-1}\sum_{i=1}^{n}{\left(x_{i}-\bar{x}\right)}^{2}}$$

where *n* is the number of measurements, *x_i_* is the output photovoltage of the ith measurement and $\bar{x}$ is the mean value of the corresponding measurement.

#### 4.9.2 SpO_2_ Monitoring During Physical Activity

All measurements were conducted with participants simultaneously wearing a medical‐grade oximeter, a commercial smartwatch, and the seedless InAs CQD‐based PPG system. Starting SpO_2_ was measured in a seated resting position, followed by two 7‐min jogging sessions with a 1‐min rest between sets. After exercise, measurement areas were wiped to remove sweat, and continuous SpO_2_ measurements were recorded from all devices at 1.5‐min intervals during recovery and release phases.

#### 4.9.3 Data Acquisition

The medical‐grade oximeter recorded SpO_2_ using its internal memory, while the smartwatch readings were manually logged from the display in real time. The raw photovoltage signals from the seedless InAs CQD‐based PPG system were monitored and stored using an oscilloscope (Keysight, DSOX1204A).

### 4.10 Mean Absolute Error Calculation

The accuracy of the measurement system was also quantified using the mean absolute error by using Equation (8).

(8) $$MAE=\frac{1}{n}\sum_{i=1}^{n}\left|y_{i}-{\hat{y}}_{i}\right|$$

where *n* is the number of measurements, *y_i_* is the reference value of the ith measurement and $\hat{y_{i}}$ is the measured value of the corresponding measurement.

#### 4.10.1 Frequency‐Dependant Photoresponse Measurement

The frequency‐dependent response of the InAs CQD photodetector was measured using a lock‐in amplifier (Stanford Research SR850, Stanford Research Systems, USA). Two lasers were tested simultaneously, each delivering a power of 0.6 µW to the active area of 0.1 cm^2^. For the 935 nm laser, the modulation frequency was first set at 0.5 kHz and sweep to 10 in 0.5 kHz increments, while maintaining the 775 nm laser modulated in 30 Hz. At each frequency, the photovoltage signal was recorded 30 times to characterize the detector's frequency‐dependent response.

#### 4.10.2 Bandwidth Measurement

The −3 dB bandwidth was determined based on the frequency at which the output signal drops to 70.7% of its low‐frequency amplitude. This corresponds to a −3 dB reduction in magnitude, defined using the voltage ratio as following Equation (9).

(9) $$20\mathrm{lo}g_{10}\;\left(\frac{V\left(f\right)}{V_{initial}}\right)=-3\;\mathrm{dB}$$

## Author Contributions

B.K.K. and S.J. contributed equally to this study. B.K.K. and S.J. developed the idea and analyzed the results. S.J. optimized the scalable synthesis process for the CQDs, synthesized the CQDs, and B.K.K. characterized the CQD ink and device. I.‐S.L. and Y.A. optimized the main and control devices. B.K.K. developed a PPG sensing platform for oximetry B.K.K. and D.K. optimized mechanism for oxygen calculation. S.P. optimized the laser fluctuation. Y.J. contributed to the manuscript writing. S.‐W.B. and I.‐H.B. supervised the project. All authors reviewed the paper and approved the final version of the manuscript.

[Correction added on 4 May 2026 after first online publication: typographical error has been updated for author “I.‐S.L.” in this version.]

## Conflicts of Interest

The authors declare no conflicts of interest.

## Supporting information




**Supporting File**: advs75303‐sup‐0001‐SuppMat.docx.
[Correction added on 4 May 2026 after first online publication: Supporting Information file has been updated in this version.]

## Data Availability

The data that support the findings of this study are available from the corresponding author upon reasonable request.
