# Supplementary material for: Seedless One‐Pot Synthesis of Colloidal InAs Quantum Dots Enabling a High‐Accuracy Photoplethysmography Oximeter
Source: Adv Sci (Weinh). 2026 Apr 20;13(38):e24375. doi: 10.1002/advs.202524375 (PMC13335444; doi:10.1002/advs.202524375)
Supplement: Supplementary file 1 — Supporting File: advs75303‐sup‐0001‐SuppMat.docx. [Correction added on 4 May 2026 after first online publication: Supporting Information file has been updated in this version.] [file ADVS-13-e24375-s001.docx]

Supporting Information

**Seedless One-pot Synthesis of Colloidal InAs Quantum Dots Enabling a High-accuracy Photoplethysmography Oximeter**

Beom Kwan Kim^1,2^, Seungin Jee^1^, Yongnam Ahn^1^, In-Suh Lee^1^, Dongeon Kim^1^, Seongchong Park^2^, Yujin Jung^3^, In-Ho Bae^2*^, Se-Woong Baek^1*^

B. K. Kim, S. Jee, Y. Ahn, I.-S. Lee, D. Kim and S.-W. Baek

^1^Department of Chemical and Biological Engineering, Korea University, Seoul, 02841 Republic of Korea

E-mail: [sewoongbaek@korea.ac.kr](mailto:sewoongbaek@korea.ac.kr)

B. K. Kim, S. Park and I.-H. Bae

^2^Division of Physical Metrology, Korea Research Institute of Standards and Science, Daejeon 305-340, Republic of Korea

E-mail: [inhobae@kriss.re.kr](mailto:inhobae@kriss.re.kr)

Y. Jung

^3^School of Chemical Engineering, Yeungnam University, Gyeongsan-Si 38541, Republic of Korea

^*^All correspondence should be addressed to I.-H. Bae and S.-W. Baek.


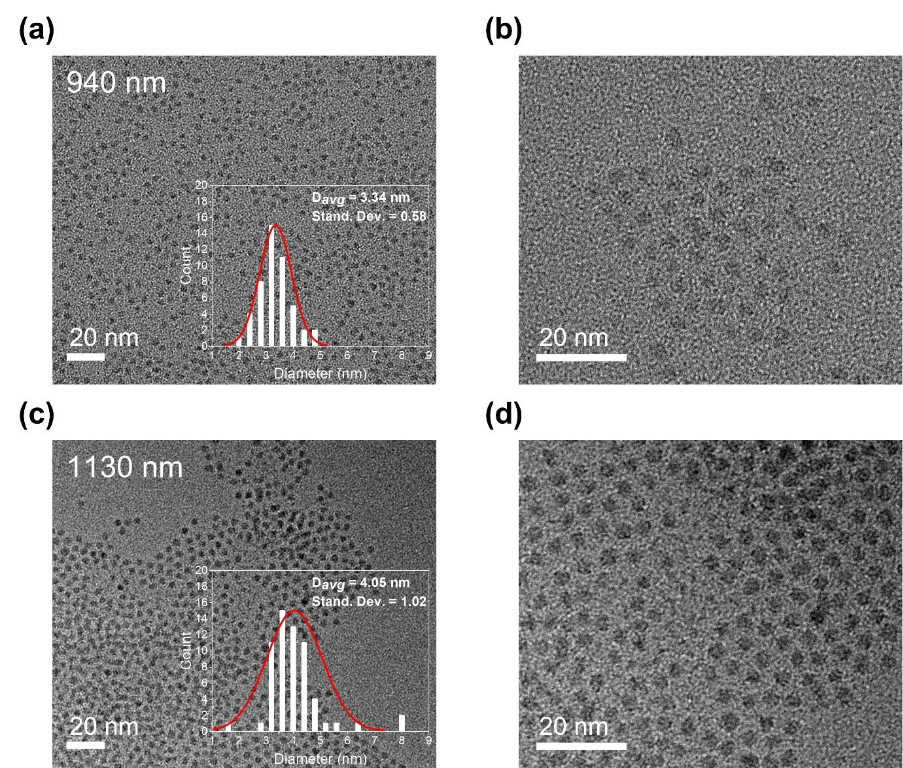


**Supplementary Fig. 1 | Size and size distribution of seedless injection InAs CQDs with different sizes.** InAs CQDs with an absorption peak at 940 nm **(a-b)** and 1130 nm **(c-d)**, with average diameters of 3.34 nm (σ = 0.58) and 4.05 nm (σ = 1.02), respectively. Scale bars: 20 nm.


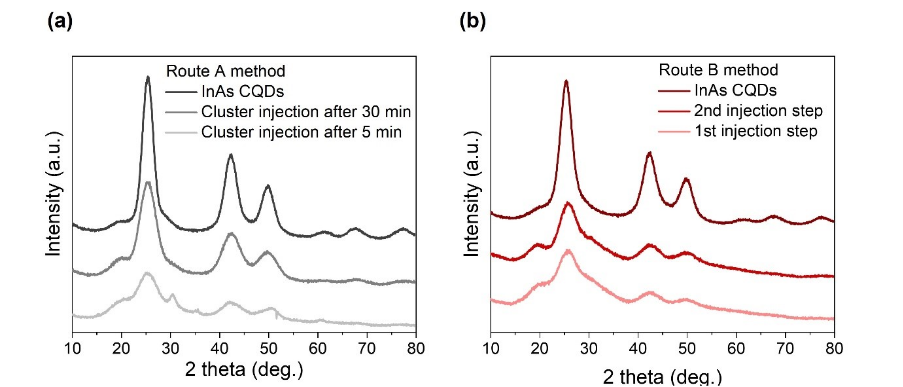


**Supplementary Fig. 2 | Comparison of the XRD patterns of InAs CQDs synthesized via different synthetic routes.** Crystal growth behavior of InAs CQDs synthesized via **(a)** Route A, as a function of increasing cluster injection time, and **(b)** Route B according to the As-stock injection steps.

**Supplementary Table 1 | XPS analysis of the atomic ratio of InAs CQDs synthesized via Routes A and B.**

| **Synthetic routes** | **Atomic ratio [%]** | | |
| --- | --- | --- | --- |
|  | **In** | **As** | **O** |
| **Route A** | 12.75 | 10.87 | 12.77 |
| **Route B** | 12.97 | 10.37 | 12.70 |


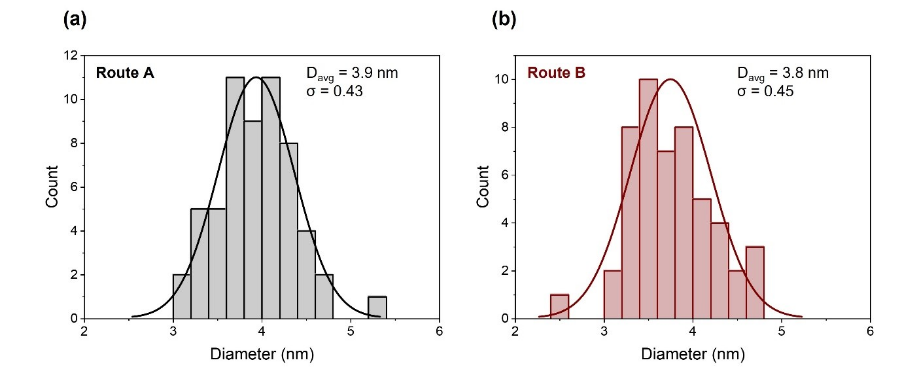


**Supplementary Fig. 3 | Size and size distribution of InAs CQDs synthesized via different synthetic routes.** **(a)** Route A-based InAs CQDs with an average diameter of 3.9 nm (σ: 0.43). **(b)** Route B-based InAs CQDs with an average diameter of 3.8 nm (σ: 0.45).

**Supplementary Table 2 | Previously reported performances of InAs CQD-based photodiodes**

| **Synthesis**  **method** | **Wavelength (nm)** | **Dark current (A/cm^2^)** | **EQE (%)** | **D* (Jones)** | **Year** | **Ref.** |
| --- | --- | --- | --- | --- | --- | --- |
| Continuous | 940 | 7.0 × 10^-7^  (-1 V) | 30 (0 V) | ~10^11^ | 2022 | 1 |
| Continuous | 950 | 2.0 × 10^-7^  (-1 V) | 37 (0V) | 1.9 × 10^11^ | 2023 | 2 |
| Continuous | 930 | 6.8 × 10^-9^ | 36 (-1 V) | 1.9 × 10^11^ | 2024 | 3 |
| Continuous | 940 | ~10^-8^ | 79 (-3 V) | 3.1 × 10^11^ | 2024 | 4 |
| **Seedless** | **960** | **8.7 × 10^-8^**  **(0 V)** | **19.6**  **(0 V)** | **3.0 × 10^11^** | **This work** | |

We benchmarked our InAs CQD photodiode against recently reported NIR InAs CQD devices operating around 930–950 nm. The previously reported devices exhibit dark current densities on the order of 10^−11^–10^−7^ A/cm^2^, EQEs of 20–79% with specific detectivities in the 10^11^ Jones range under different reverse biased conditions. In comparison, our device shows a dark current of 8.7 × 10^−8^ A/cm^2^ and an EQE of 19.6% at 0 V, corresponding to a specific detectivity (*D*^*^) of 3.0 × 10^11^ Jones at 960 nm. These values fall within the performance range reported for NIR InAs CQD photodiodes.


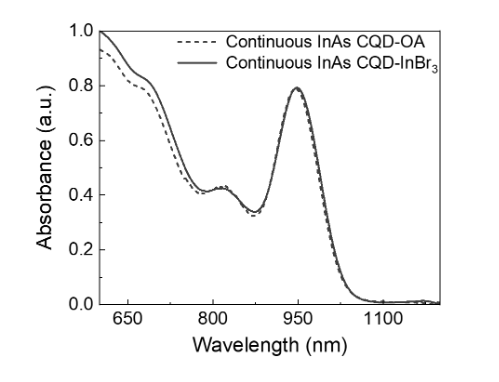


**Supplementary Fig. 4 |** **UV-vis spectroscopy of continuous InAs CQD ink before (dash) and after (solid) InBr_3_ ligand exchange.** After ligand exchange, a red shift of ~3 nm is observed, indicating effective InBr₃ surface passivation, consistent with seedless injection InAs CQD inks.


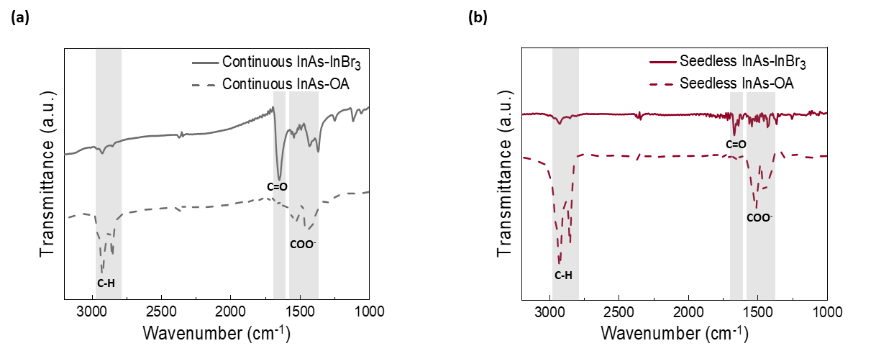


**Supplementary Fig. 5 |** **FT-IR spectroscopy comparison of continuous (gray) and seedless(red) InAs CQD before (dash) and after (solid) InBr_3_ ligand exchange in pellets.** OA-passivated InAs CQDs exhibit intensity peaks for CH_3_ stretching vibrations^1,3,5^ at 2800–2900 cm^-1^ and carboxyl groups at 1750 cm^-1^. After InBr_3_ ligand exchange, these peaks disappeared, indicating successful surface modification. Similar spectral changes are observed for both continuous and seedless InAs CQDs, confirming that the surface modification is consistently achieved in the seedless CQDs.


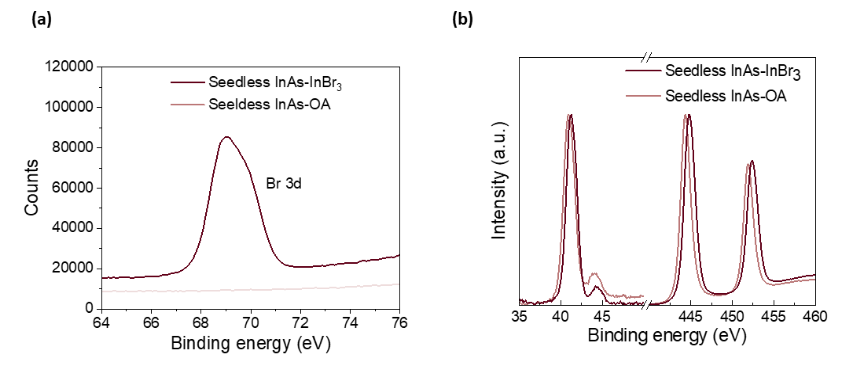


**Supplementary Fig. 6 |** **X-ray photoelectron spectroscopy (XPS) of seedless InAs CQD films before (red) and after InBr_3_ ligand exchange (pink).** XPS spectra showing a significant increase in the **(a)** Br 3d signal at 68–70 eV after ligand exchange, indicating the successful substitution of bromide ligands compared with pristine InAs CQDs^1,3^. The overall XPS profile exhibits a **(b)** slight red-shift, reflecting changes in the chemical environment and charge state consistent with effective ligand exchange and surface passivation^1,3^.


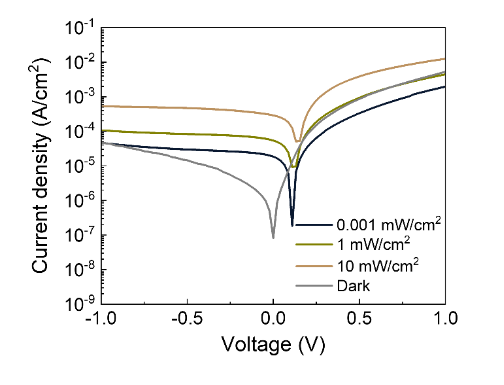


**Supplementary Fig. 7 |** ***J*–*V* curves of continuous InAs CQD-based photodetectors.** Continuous InAs CQD-based photodetector exhibits 0.132 V using 1.0 ND filtered. This result indicates that seedless synthesized InAs CQD exhibits similar performance for leakage, carrier extraction control to continuous InAs CQD based photodetector.


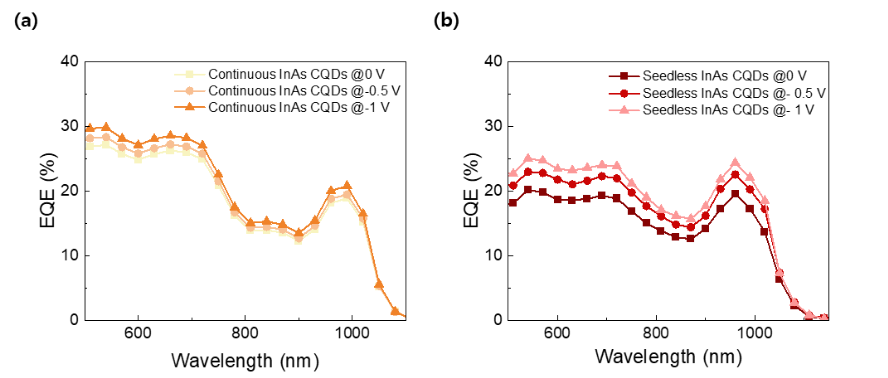


**Supplementary Fig. 8 |** **External quantum efficiency (EQE) measurements of continuous and seedless InAs CQD-based photodetectors under a biased condition. (a)** Continuous InAs CQDs exhibited an EQE of 20.4% at -1 V. **(b)** Seedless InAs CQDs exhibited a higher EQE of 24.4% under the same bias condition.


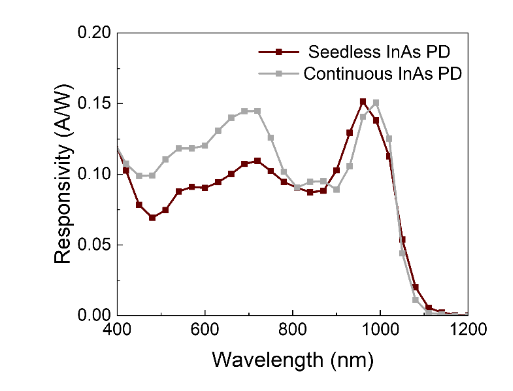


**Supplementary Fig. 9 |** **Responsivity of continuous and seedless InAs CQD-based photodetectors.** Continuous (gray) and seedless (red) InAs CQD PDs exhibit similar responsivity of ~0.15 A/W at 950 and 960 nm, indicating comparable optical performance across this spectral range.


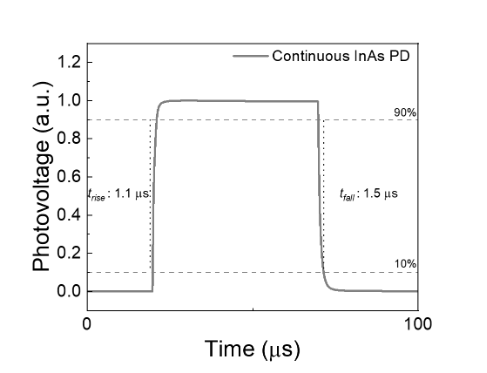


**Supplementary Fig. 10** **|** **Rise and fall time of the continuous InAs CQD-based photodetector.** The continuous InAs CQD device exhibits comparable rise (1.1 μs) and fall (1.5 μs) times under 935 nm laser illumination, with the seedless InAs CQD device.

**Supplementary Table 3. Winterbourn quantification of Hb/HbO_2_ sample preparation**

| **Preparation sample** | **HbO₂ (a.u.)** | **Hb (a.u.)** | **SpO₂(%)** |
| --- | --- | --- | --- |
| **95** | 0.067577 | 0.003646 | **94.8811** |
| **96** | 0.068377 | 0.002846 | **96.0045** |
| **97** | 0.069045 | 0.002178 | **96.9423** |
| **98** | 0.070009 | 0.001214 | **98.2957** |
| **99** | 0.070469 | 0.000754 | **98.9414** |


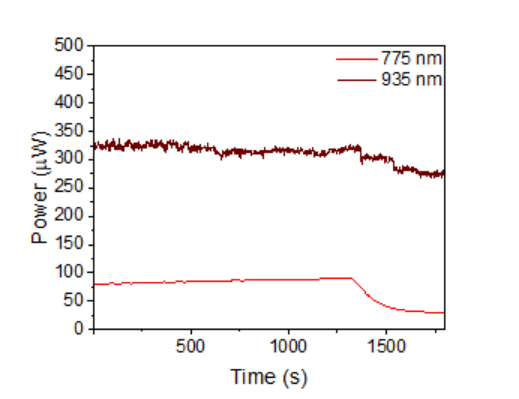


**Supplementary Fig. 11 | Power fluctuation of the red and infrared** l**aser diodes over the operation time.** The decrease in emission intensity after 1300 s indicates the inherent power degradation of the 775 nm (bottom, red) and 935 nm (top, brown) laser diodes during continuous operation. While the output of the 935 nm laser exhibits slight noise and minimal degradation, allowing the signal to remain stable, the 775 nm laser output decreases by 30% after 1300 s. This can lead to signal-level fluctuations and directly affects the detection accuracy.

**Supplementary Table 4** **|** Previously reported commercial PPG oximeter with red/green and infrared light emitters

|  | Model | Detector | MAE (%) | Maximum error margin (%) | Sampling rate  (kHz) | Reference |
| --- | --- | --- | --- | --- | --- | --- |
| 1 | Apple watch 7 | Si | **2.2** | 4.99 | **-** | 6 |
| 2 | Garmin Venus 2S | Si | **5.8** | 6.7 | **-** | 6 |
| 3 | Galaxy watch 5 | Si | **3.5** | 3.8 | 0.1 | 7 |
| 4 | Strap type | MAX  30102 | **2.1** | 3.3 | 1 | 8 |
| 5 | **InAs CQD** | | **1.43** | **1.98** | **2.5** | **Present study** |

1. MAE: Mean absolute error

**References**

1 Sun, B. *et al.* Fast Near-Infrared Photodetection Using III-V Colloidal Quantum Dots. *Adv Mater* **34,** 2203039 (2022).
<https://doi.org/10.1002/adma.202203039>

2 Xia, P. *et al.* Sequential Co-Passivation in InAs Colloidal Quantum Dot Solids Enables Efficient Near-Infrared Photodetectors. *Adv Mater* **35**, e2301842 (2023). <https://doi.org/10.1002/adma.202301842>

3 Si, M. J. *et al.* Colloidal InAs Quantum Dot-Based Infrared Optoelectronics Enabled by Universal Dual-Ligand Passivation. *Adv Sci* **11**, 2306798 (2024). <https://doi.org/10.1002/advs.202306798>

4 Jiang, L. *et al.* High-performance near-infrared fluorescence probe for fast and specific visualization of harmful sulfite in food, living cells, and zebrafish. *Chem Eng J* **427**, 131563 (2022).
<https://doi.org/10.1016/j.cej.2021.131563>

5 Jee, S. *et al.* P-Type Colloidal InSb Quantum Dot Ink Enables III-V Group Bulk-Heterojunction Shortwave Infrared (SWIR) Photodetector. *Adv Opt Mater* **12,** 2303097 (2024).
<https://doi.org/10.1002/adom.202303097>

6 Jiang, Y. *et al.* Investigating the accuracy of blood oxygen saturation measurements in common consumer smartwatches. *PLOS Digit Health* **2**, e0000296 (2023).
<https://doi.org/10.1371/journal.pdig.0000296>

7 Walzel, S. *et al.* Evaluation of Leading Smartwatches for the Detection of Hypoxemia: Comparison to Reference Oximeter. *Sensors (Basel)* **23**, 9164 (2023).
<https://doi.org/10.3390/s23229164>

8 Longmore, S. K. *et al.* A comparison of reflective photoplethysmography for detection of heart rate, blood oxygen saturation, and respiration rate at various anatomical locations. *Sensors-Basel* **19**, 1874 (2019).
<https://doi.org/10.1016/j.jacc.2023.04.054>
